# Increased Susceptibility to Dextran Sulfate Sodium-Induced Colitis in the Endoplasmic Reticulum Stress Transducer OASIS Deficient Mice

**DOI:** 10.1371/journal.pone.0088048

**Published:** 2014-02-03

**Authors:** Kenta Hino, Atsushi Saito, Rie Asada, Soshi Kanemoto, Kazunori Imaizumi

**Affiliations:** Department of Biochemistry, Institute of Biomedical & Health Sciences, University of Hiroshima, 1-2-3 Kasumi, Minami-ku, Hiroshima, Japan; St. Jude Children's Hospital, United States of America

## Abstract

OASIS is a basic leucine zipper (bZIP) transmembrane transcription factor that is activated in response to endoplasmic reticulum (ER) stress. Previously, we showed that OASIS regulates final maturation of goblet cells in the large intestine. In the present study, to elucidate the roles of OASIS under pathophysiological conditions, we examined the stress response and inflammatory responses in *Oasis* deficient (*Oasis*−/−) mice exposed to dextran sulfate sodium (DSS) to induce colitis. A significant loss of body weight and an increase of mortality were observed in *Oasis*−/− mice with DSS-induced colitis compared with those in WT mice. The mucosa of the large intestine in *Oasis*−/− mice exhibited severe damage involving inflammatory cell infiltration. The expression levels of ER stress and apoptosis markers in intestinal epithelial cells were upregulated in *Oasis*−/− mice. These abnormalities were improved by treatment with tauroursodeoxycholic acid, a chemical chaperone that facilitates protein folding. Taken together, our findings demonstrate that OASIS plays important roles in protection of the large intestinal mucosa in DSS-induced colitis through attenuation of ER stress and inflammation.

## Introduction

The endoplasmic reticulum (ER) is a cellular organelle that regulates protein synthesis, modification, and folding. Various genetic and environmental insults lead to accumulation of unfolded or misfolded proteins in the ER. These conditions are collectively termed as “ER stress”. Excessive ER stress and/or prolonged ER stress cause cellular damage followed by induction of apoptosis. When cells are exposed to ER stress, three major ER stress transducers, PKR-like ER kinase (PERK) [1], inositol-requiring kinase 1 (IRE1) [2], [3], and activating transcription factor 6 (ATF6) [4], sense the accumulation of unfolded proteins and transduce signals to alleviate ER stress. This stress response is called the unfolded protein response (UPR) [5], [6]. In addition to these canonical ER stress transducers, there are novel types of ER stress transducers. These novel transducers share a region of high sequence similarity with ATF6 and have been named the OASIS family [7] including OASIS/CREB3L1 [8], BBF2H7/CREB3L2 [9], CREBH/CREB3L3 [10], CREB4/CREB3L4 [11], and Luman/CREB3 [12]. Recent studies have demonstrated that OASIS family members are involved in the regulation of cell differentiation and maturation, and the maintenance of cellular homeostasis [13]–[21].

*Oasis* was originally identified as a gene expressed in long-term cultures of astrocytes [8]. Under normal conditions, OASIS localizes to the ER membrane. When cells are exposed to ER stress, OASIS is cleaved at the transmembrane region, and its processed N-terminal fragments containing a basic leucine zipper (bZIP) domain are translocated into the nucleus to promote transcription of target genes [22]–[24]. OASIS is also strongly expressed in goblet cells of the large intestine. Infant *Oasis* deficient (*Oasis*−/−) mice exhibit abnormalities of goblet cell differentiation and maturation in their large intestine [25].

Inflammatory bowel disease (IBD) is a refractory intestinal disease characterized by chronic inflammation involving mucosal erosion and ulcers in parts of the gastrointestinal tract. Moreover, IBD is induced by continuous immune responses to microbial antigenic stimulation [26] or impaired secretion of mucus from goblet cells [27]. However, the detailed mechanisms of this disease are still unclear. Recent studies have shown that ER stress is induced in the large intestinal mucosa of patients with IBD such as Crohn's disease (CD) and ulcerative colitis (UC) [28]–[31]. Additionally, primary genetic abnormalities have been found in *Xbp1*, which is associated with UPR, and in *Agr2* and *Ormdl3* that are induced by UPR signaling [31]–[35]. Experimentally, *Ire1β*−/− and *Atf6α*−/− mice treated with dextran sulfate sodium (DSS), which is cytotoxic to intestinal epithelial cells and widely accepted to induce colitis in mouse models, show early development of colitis [36], [37]. Furthermore, *Xbp1*−/− mice show an increased susceptibility to DSS-induced colitis [31]. These previous studies indicate that ER stress and its stress responses may be related to the development of IBD.

In the present study, we examined the susceptibility and inflammatory responses to DSS-induced colitis in *Oasis*−/− mice, and found the possibility that OASIS plays crucial roles in suppression of ER stress and inflammation in DSS-induced colitis through mucus production.

## Materials and Methods

### Mice

*Oasis*−/− mice were previously established in our laboratory [38]. In all studies comparing adult wild-type (WT) and *Oasis*−/− mice, we used sex-matched littermates derived from mating of *Oasis*+/− mice. The experimental procedures and housing conditions for the animals were approved by the Committee of Animal Experimentation, Hiroshima University. For non-survival studies, mice were sacrificed by cervical dislocation under anesthetic condition using diethyl ethel, and all efforts were made to minimize suffering after administration of 3.5% DSS in the drinking water for 5 days. For survival study, the mice were checked each day for morbidity and weight was recorded after administration of 3.5% DSS in the drinking water. Moribund animals were sacrificed using humane endpoints. The mice showed behavioral signs unresponsive to appropriate intervention including sunken eyes, hunched posture, piloerection/matted fur, one or more unresolving skin ulcers, and abnormal vocalization when handled were judged as moribund, and immediately sacrificed using the above method. Non-moribund animals that died during the course of the survival curve died as a direct result of the DSS.

### DSS-induced colitis and drug administration

Adult mice were administered with 3.5% DSS (MP Biomedicals) in sterilized water for 5 or 10 days to induce acute colitis. Tauroursodeoxycholic acid (TUDCA) (10 mg/ml, sodium salt; Wako) in sterilized PBS was administered orally (160 mg/kg/day) for 5 days.

### Cell culture

LS174T cells (derived from colon, an immortal non-transformed cell line from a human donor) were obtained from American Type Culture Collection (ATCC) and cultured in Eagle's minimal essential medium supplemented with 10% fetal calf serum and 1% non-essential amino acids. Tunicamycin (Tm; 0.6 µg/mL) and TUDCA (0.2 mg/ml) were used for cell treatments.

### Histological analysis

Large intestines of adult mice were fixed overnight in 10% formalin (Wako). Then, the samples were dehydrated with ethanol, embedded in paraffin, and sectioned (5 µm). Hematoxylin-eosin (HE) and periodic acid schiff (PAS) staining were performed according to standard protocols. PAS-positive cells were manually counted under a microscope (BX51; Olympus), and PAS-positive areas were quantitated by Image J software (NIH). HE-stained colon sections were pathologically scored as described previously [39].

### Histological scoring

Tissues from WT and *Oasis*−/− mice with DSS-induced colitis were stained with HE. The sections were scored in a blinded manner. The scoring criteria were based on a previous study [39].

### *In situ* hybridization

*In situ* hybridization was performed using digoxigenin-labeled *Bip* and *Chop* cRNA probes. Antisense probes were prepared by *in vitro* transcription in the presence of digoxigenin-labeled dUTP using various cDNAs subcloned into pGEM-Teasy vectors (Promega) as templates. Deparaffinized sections were washed with PBS and then treated with 0.1% proteinase K for 10 min. After washing with PBS, the sections were re-fixed for 20 min with 4% formalin in PBS and then treated with 0.1 M triethanolamine and 2.5% anhydrous acetic acid in diethylpyrocarbonate (DEPC)-treated water for 10 min followed by washing with PBS. The sections were prehybridized for 1 h at 37°C in hybridization buffer (0.01% dextran sulfate, 0.01 M Tris-HCl, pH 8.0, 0.05 M NaCl, 50% formamide, 0.2% sarcosyl, 1× Denhardt's solution, and 0.2 mg/ml salmon testis DNA) and then hybridized overnight at 55°C in hybridization solution with 100 ng/ml cRNA probe. After washing with 4×saline sodium citrate (SSC) buffer (1×SSC: 0.15 M NaCl and 0.015 M sodium citrate, pH 7.0) for 20 min at 60°C, the sections were washed with 2×SSC buffer and 50% formamide in DEPC-treated water for 30 min at 60°C. Sections were treated RNase A in RNase buffer (10 mM Tris-HCl, pH 7.4, 1 mM EDTA (pH 8.0), and 0.5 M NaCl) for 15 min at 37°C to remove un-hybridized probes. After RNase treatment, the sections were washed with 2×SSC buffer and 50% formamide in DEPC-treated water for 30 min at 60°C and then treated with 1.5% blocking reagent (Roche) in 100 mM Tris-HCl (pH 7.5) and 150 mM NaCl for 1 h at room temperature. To detect digoxigenin-labeled cRNA probes, an anti-digoxigenin antibody conjugated to alkaline phosphatase (Roche) was used at a dilution of 1∶500, and then the color was developed by incubation in a solution of 4-nitro blue tetrazolium chloride (Wako) and 5-bromo-4-chloro-3-indolyl phosphate (Roche).

### TUNEL staining

Large intestines of adult mice were fixed overnight in 4% paraformaldehyde. The samples were then dehydrated with ethanol, embedded in paraffin, and sectioned (5 µm). TUNEL staining was performed using the DeadEnd Fluorometric TUNEL system (Promega) according to the manufacturer's protocol. Sections were visualized under a fluorescence microscope.

### RNA isolation and RT-PCR

Total RNA was isolated from LS174T colonic epithelial cells and various tissues using ISOGEN (Wako) according to the manufacturer's protocol. Preparation of colonic epithelial cells from the large intestine of adult mice was performed using a previously published protocol [36] with some modification. Briefly, the colon was removed and washed with solution A (96 mM NaCl, 27 mM sodium citrate, 1.5 mM KCl, 0.8 mM KH_2_PO_4_, and 5.6 mM Na_2_HPO_4_ 12H_2_O in sterilized water, pH 7.4). Square pieces of tissue were placed in 10 ml of solution A at 37°C for 10 min with gentle shaking. The tissue fragments were then incubated in 5 ml of solution B (0.1 mM EDTA, 115 mM NaCl, 25 mM NaHCO_3_, 2.4 mM K_2_HPO_4_, 0.4 mM KH_2_PO_4_, and 2.5 mM glutamine in sterilized water, pH 7.4) at 37°C for 30 min with gentle shaking. Digestion was stopped by addition of 100 mM CaCl_2_. Messenger RNA was purified from total RNA of DSS-treated large intestinal epithelia using an Oligotex-dT30 Super mRNA Purification kit (Takara) according to the manufacturer's protocol. First-strand cDNA was synthesized in a 20 µl reaction volume using a random primer (Takara) and Moloney murine leukemia virus reverse transcriptase (Invitrogen). PCR was performed using each specific primer set in a total volume of 30 µl containing 0.8 µM of each primer, 0.2 mM dNTPs, 3 U *Taq* polymerase, and 10× PCR buffer (Agilent). The PCR products were subjected to electrophoresis on a 4.8% acrylamide gel. RT-PCR analysis was performed in a semi-quantitative manner. The density of each band was quantified using Adobe Photoshop Elements 2.0 (Adobe Systems). The primer sets are listed in Supplemental Table S1.

### Western blotting

Proteins were extracted from large intestines and epithelial cells using cell extraction buffer containing 10% SDS, 0.5 M EDTA (pH 8.0), 100 mM methionine, and a protease inhibitor mixture (Calbiochem). Preparation of colonic epithelial cells was carried out as described in “RNA isolation and RT-PCR”. The lysates were incubated on ice for 45 min. After centrifugation at 16,000×*g* for 15 min, the protein concentrations of the supernatants were determined by a BCA assay kit (Thermo Scientific). Equal amounts of proteins were subjected to sodium dodecyl sulfate-polyacrylamide gel electrophoresis. For immunoblotting, the following antibodies and dilutions were used: anti-β-actin (1∶3000; Sigma), anti-OASIS (1∶1000) (37), anti-cleaved caspase-3 (1∶1000; Cell signaling), and anti-caspase-12 (1∶1000; Cell signaling). The density of each band was quantified using Adobe Photoshop Elements 2.0.

### Luciferase assay

p-Luc nuclear factor (NF)-κB was kindly provided by Y. Tsuchiya (Hiroshima University). LS174T cells were transfected with 0.2 µg/µl p-Luc NF-κB and 0.02 µg/µl pRL-TK using Lipofectamine 2000 (Invitrogen). After 24 h, luciferase activities were measured using the Dual-Luciferase Reporter Assay System (Promega) and GloMax Multi+ Detection System (Promega) according to the manufacturer's protocol. Relative activities were defined as the ratio of firefly luciferase activity to renilla luciferase activity.

### Statistical analysis

Statistical comparisons were made using the unpaired Student's *t*-test. P-values of less than 0.05 were considered statistically significant.

## Results

### The number of mature goblet cells is decreased in adult *Oasis*−/− mice

**Figure 1:**
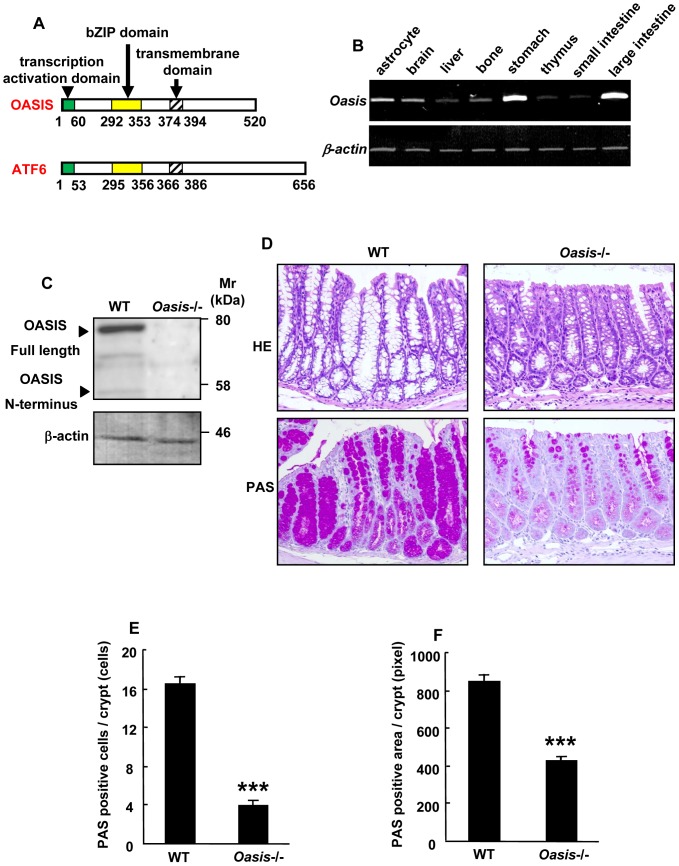
Decreased number of mature goblet cells in the large intestine of adult *Oasis*−/− mice. (A) Peptide features of mouse OASIS and ATF6. (B) RT-PCR analysis of *Oasis* mRNA in various tissues from 8-week-old mice. *Oasis* mRNA was highly expressed in the large intestine. (C) Western blotting of OASIS in the large intestine from 8-week-old mice. Both full-length OASIS (full OASIS) and the N-terminal OASIS (N-OASIS) were detected in the large intestine. (D) HE (upper panels) and PAS (lower panels) staining of the large intestines from 8-week-old WT and *Oasis*−/− mice. In the *Oasis*−/− large intestine, the number of mature goblet cells containing large mucosal granules was markedly decreased compared with that in WT mice. (E and F) Quantification of (E) PAS-positive cells and (F) areas of PAS-positive cells in (D) (*n* = 9). Values represent the means ± s.d. ***P<0.001.

OASIS is structurally similar to ATF6, which is one of the ER stress transducers, and contains a transcription-activation domain, bZIP domain and transmembrane domain (Fig. 1A). The expression of OASIS mRNA was detected in the brain, bone, and digestive system (Fig. 1B). Among them, the strongest signals were observed in the large intestine. We previously reported that differentiation from immature to mature goblet cells is impaired in infant *Oasis*−/− mice [25]. Western blotting showed that both full-length OASIS and its N-terminus (the active form) were also robustly expressed in the large intestine of adult mice (8-weeks-old) (Fig. 1C). Next, we performed morphological analyses of the large intestine in 8-week-old *Oasis*−/− mice. HE staining showed a marked decrease in the number of mature goblet cells containing large vacuoles in *Oasis*−/− large intestinal mucosa compared with that in WT mice. The number of PAS-positive goblet cells and the intracellular granules were reduced in *Oasis*−/− mice (Fig. 1D–F). These results indicate that the number of mature goblet cells containing abundant mucus is also significantly decreased in the adult *Oasis*−/− large intestinal mucosa as well as in infant *Oasis*−/− mice.

### *Oasis*−/− mice exhibit increased susceptibility to DSS-induced colitis

**Figure 2:**
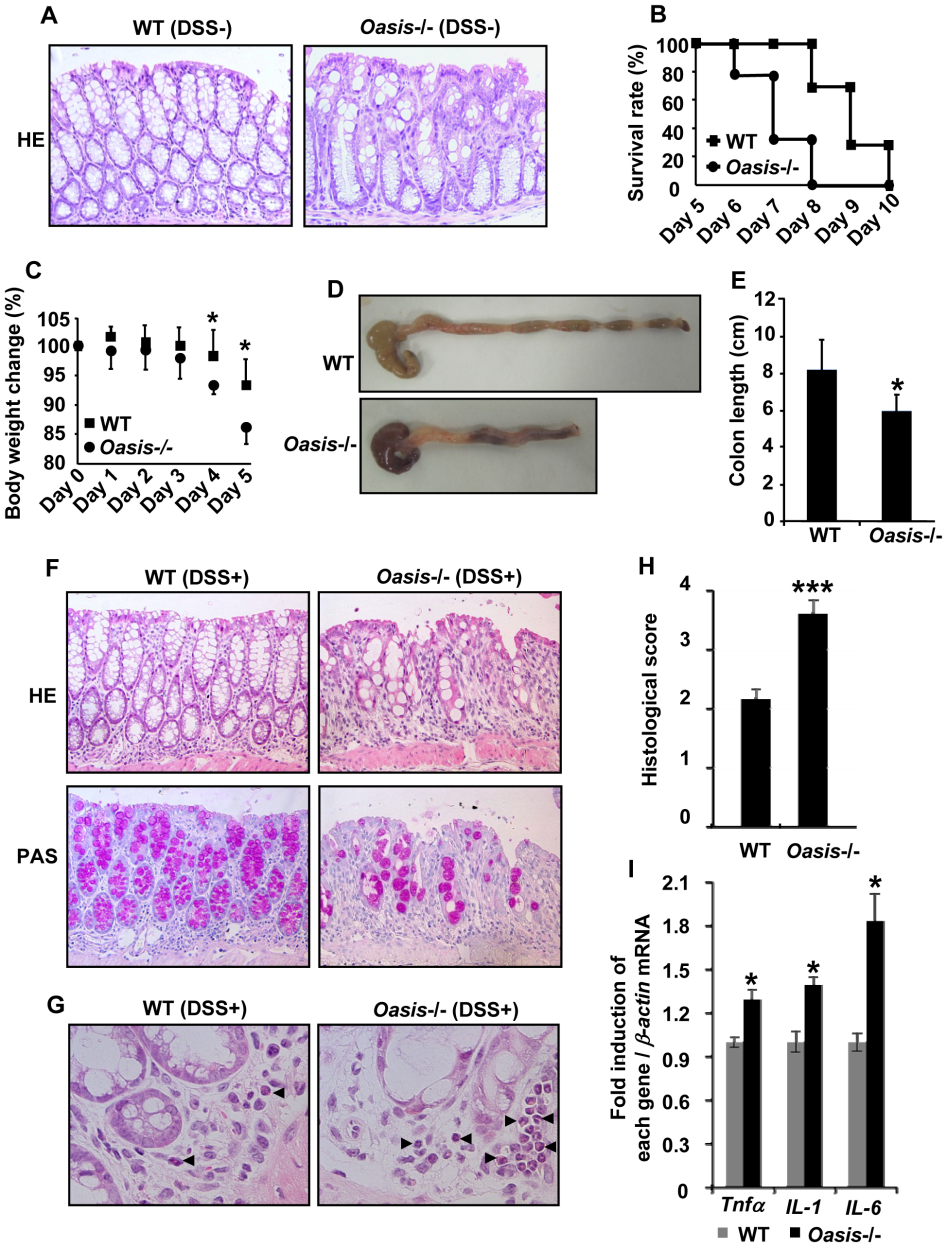
Increased susceptibility to DSS-induced colitis in *Oasis*−/− mice. (A) HE staining of the large intestines from 12-week-old WT and Oasis−/− mice. (B) Survival rates of WT and *Oasis*−/− mice that received 3.5% DSS for 10 days. Note that the mortality of *Oasis*−/− mice was observed at 2 days earlier than that of WT mice (*n* = 9). (C) Body weight changes of WT and *Oasis*−/− mice. After administration of DSS, *Oasis*−/− mice showed severe loss of body weight compared with that of WT mice (*n* = 9). (D) WT and *Oasis*−/− large intestines exposed to 3.5% DSS for 5 days. The large intestine of *Oasis*−/− mice exposed to 3.5% DSS was shortened and bleeding, and there was a reduction in the number of fecal pellets. (E) Quantification of colon length in (D) (*n* = 7). (F) HE (upper panels) and PAS (lower panels) staining of large intestines from WT and *Oasis*−/− mice exposed to 3.5% DSS for 5 days. Note that the *Oasis*−/− large intestine exhibited mucosal damage, degeneration of the mucosal epithelium, a decrease in the number of goblet cells, and an increase of crypt loss compared with those in WT large intestines. (G) Higher magnification of HE staining in (F). Arrowheads show inflammatory cells. The lamina propria of the *Oasis*−/− large intestine showed severe infiltration of inflammatory cells including macrophages and neutrophils. (H) Histological scores of colitis in WT and *Oasis*−/− mice that received 3.5% DSS for 5 days (*n* = 6). The scoring was carried out as described in the EXPERIMENTAL PROCEDURES. (I) RT-PCR analysis of inflammatory cytokines in WT and *Oasis*−/− large intestinal mucosa exposed to 3.5% DSS for 5 days (*n* = 5). The expression levels of inflammatory cytokines were higher in *Oasis*−/− mice than those in WT mice. Values represent the means ± s.d. *P<0.05; ***P<0.001.

The knockout mouse of *Muc2*, which is one of the major components of mucus, has revealed mucin depletion and development of spontaneous colitis [27]. However, in *Oasis*−/− mice that exhibit impaired maturation of goblet cells, spontaneous colitis was not observed until 12 weeks of age (Fig. 2A). We next examined the effects of DSS, a toxin of mucosal epithelial cells [40], on inflammatory responses in *Oasis*−/− mice. 12-week-old WT and *Oasis*−/− mice received 3.5% DSS in their drinking water for 5 or 10 days. The mortality of *Oasis*−/− mice after 3.5% DSS exposure for 10 days was observed at approximately 2 days earlier than that of WT mice (Fig. 2B), and the mice showed severe loss of body weight by day 5 (Fig. 2C). Moreover, the large intestine in *Oasis*−/− mice was shorter and showed severe bleeding, and a reduction in the number of fecal pellets (indicating diarrhea) compared with that in WT mice after administration of 3.5% DSS for 5 days (Fig. 2D, E). Pathologically, the *Oasis*−/− large intestine exhibited mucosal damage involving degeneration of the mucosal epithelium and a decrease in the number of goblet cells together with crypt loss (Fig. 2F). Furthermore, severe infiltration of inflammatory cells, including mononuclear cells and neutrophils, was observed in lesions of the lamina propria in *Oasis*−/− mice (Fig. 2G). Next, we evaluated the histopathology of mice with DSS-induced colitis by histological scoring. The scores from 0 to 4 were based on a previous study [39] as follows. No changes from normal tissue (grade 0). A few multifocal mononuclear cells had infiltrated into the lamina propria accompanied by minimal epithelial hyperplasia and no depletion of mucus (grade 1). Lesions were more frequent and several inflammatory cells had infiltrated into the lamina propria (grade 2). Mild epithelial hyperplasia, small epithelial erosions, mucin depletion, and the lesions were more frequent than grade 2 (grade 3). Lesions, inflammatory cells, and crypt abscesses were observed occasionally, moderate epithelial hyperplasia and mucin depletion were observed, the lesions were more severe than grade 3 lesions (grade 4). The average score was higher in *Oasis*−/− mice (3.60) than that in WT mice (2.12) (Fig. 2H). RT-PCR analysis of mRNA prepared from the large intestinal mucosa of *Oasis*−/− mice exposed to 3.5% DSS for 5 days showed significant increases in the expression levels of *tumor necrosis factor-α* (*Tnfα*), *interleukin-1* (*IL-1*), and *interleukin-6* (*IL-6*) compared with WT mice (Fig. 2I). Taken together with the pathological findings, we found that epithelial damage and inflammation are aggravated in the *Oasis*−/− large intestine exposed to DSS.

### Acceleration of ER stress in *Oasis*−/− mice with DSS-induced colitis

**Figure 3:**
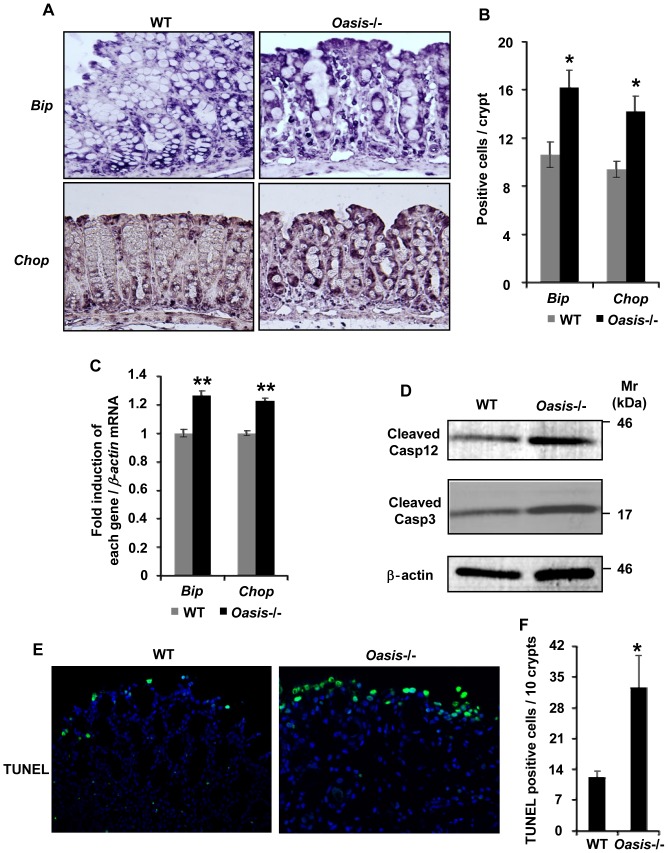
ER stress is accelerated in *Oasis*−/− mice with DSS-induced colitis. (A) *In situ* hybridization of ER stress markers *Bip* (upper panels) and *Chop* (lower panels) in the large intestinal mucosa of WT and *Oasis*−/− mice exposed to 3.5% DSS for 5 days. In WT mice, both ER stress markers were mainly observed in the basal crypt. In contrast, these markers were expressed in both the basal and apical crypts of *Oasis*−/− mice. (B) The number of *Bip*- and *Chop*-positive cells per crypt in (A) (*n* = 5). The number of cells positive for each ER stress marker was increased by about 1.5-fold in the *Oasis*−/− large intestinal mucosa compared with that in WT mice. (C) RT-PCR analysis of ER stress markers *Bip* and *Chop* in the large intestine of WT and *Oasis*−/− mice exposed to 3.5% DSS for 5 days (n = 5). (D) Western blotting of cleaved caspase-12 and -3 in the large intestinal mucosa of WT and *Oasis*−/− mice exposed to 3.5% DSS for 5 days. (E) TUNEL staining of the large intestinal mucosa in WT and *Oasis*−/− mice exposed to 3.5% DSS for 5 days. (F) The number of TUNEL-positive cells per 10 crypts in (E) (n = 4). The number of TUNEL-positive cells was increased in the apical portion of the mucosa in *Oasis*−/− mice. Values represent the means ± s.d. *P<0.05.

Previous studies have reported that ER stress is accelerated in patients with IBD (CD and UC) [28]–[31]. Furthermore, genomic deletion of *Xbp1* or *Ire1β*, which are both ER stress-related genes, increases susceptibility to DSS-induced colitis [31], [36]. Next, we investigated the expression of ER stress markers *Bip* and *Chop* by *in situ* hybridization in the large intestinal tissues of WT and *Oasis*−/− mice exposed to 3.5% DSS for 5 days (Fig. 3A). In WT mice, the signals of the ER stress markers were mainly observed in epithelial cells of the basal portions of crypts. These signals were more intense and detected in both the basal and apical portions of crypts in the *Oasis*−/− large intestine. The number of *Bip*- and *Chop*-positive cells and the expression levels of those genes in the *Oasis*−/− large intestine were higher than those in the WT large intestine (Fig. 3B, C). These results indicate that ER stress is enhanced in the large intestine of *Oasis*−/− mice exposed to DSS. To examine the relationship between the enhanced ER stress and cell death of epithelial cells in the large intestine of *Oasis*−/− mice, we performed western blotting of caspase-12 and -3 (Fig. 3D). Both cleaved caspase-12 and -3 were detected in WT and *Oasis*−/− large intestinal mucosae, but their amounts were relatively higher in *Oasis*−/− mice. Furthermore, a large number of TUNEL-positive cells was observed in the apical portions of crypts in the *Oasis*−/− large intestine (Fig. 3E, F). These findings suggest that severe damage of the large intestinal mucosa in *Oasis*−/− mice may be caused by acceleration of ER stress-induced apoptosis.

### Suppression of ER stress attenuates colon injury

**Figure 4:**
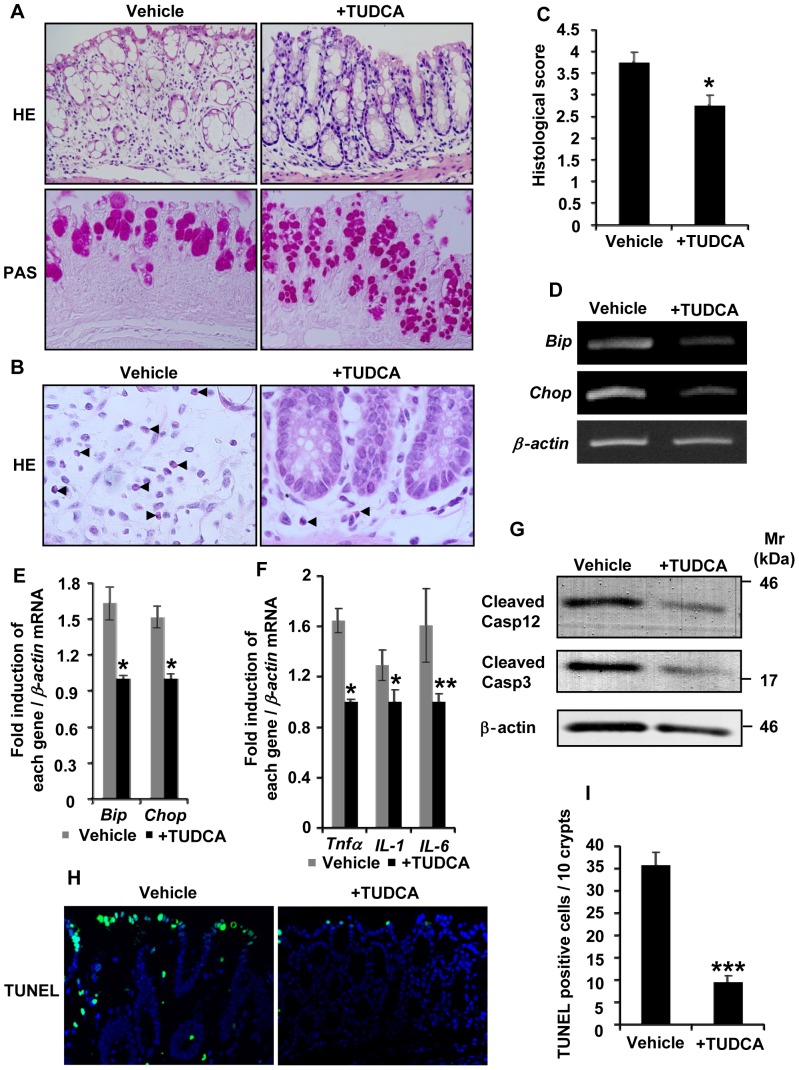
TUDCA alleviates DSS-induced colitis. All the *Oasis*−/− mice received 3.5% DSS and some were given TUDCA (+TUDCA) and others were given the same volume of PBS (vehicle) daily by oral administration for 5 days. (A) HE (upper panels) and PAS (lower panels) staining of the large intestinal mucosa of *Oasis*−/− mice exposed to 3.5% DSS and TUDCA or the vehicle. (B) Higher magnification of HE staining in (A). Arrowheads show inflammatory cells. (C) Histological scores of control and TUDCA-treated *Oasis*−/− mice that received 3.5% DSS (*n* = 4). The pathological findings were markedly improved in *Oasis*−/− mice treated with TUDCA. (D) RT-PCR analysis of *Bip* and *Chop* in the large intestinal mucosa of *Oasis*−/− mice. The expression levels of these ER stress markers in the large intestinal mucosa of *Oasis*−/− mice were decreased by treatment with TUDCA. (E) Quantification of the expression levels of *Bip* and *Chop* in (D) (*n* = 4). (F) RT-PCR analysis of inflammatory cytokines in the large intestinal mucosa of *Oasis*−/− mice exposed to 3.5% DSS and TUDCA or the vehicle for 5 days (*n* = 4). Note that the expression levels of inflammatory cytokines were decreased in *Oasis*−/− mice that received TUDCA. (G) Western blotting of cleaved caspase-12 and -3 in *Oasis*−/− large intestinal mucosa exposed to 3.5% DSS and TUDCA or the vehicle for 5 days. (H) TUNEL staining of the large intestinal mucosa in *Oasis*−/− mice that received 3.5% DSS and TUDCA or the vehicle. (I) The number of TUNEL-positive cells in (H) (n = 4). The number of TUNEL-positive cells was decreased in *Oasis*−/− mice treated with TUDCA. Values represent the means ± s.d. *P<0.05; **P<0.01.

Next, we examined whether treatment with TUDCA, which is commonly used as a chemical chaperone to alleviate ER stress [37], suppressed the damage in the large intestine of *Oasis*−/− mice exposed to DSS. *Oasis*−/− mice exposed to DSS were treated with TUDCA (160 mg/kg body weight) or the vehicle (PBS) by oral administration daily for 5 days. *Oasis*−/− mice treated with TUDCA showed very mild epithelial injury in their crypts, and the number and morphology of crypts were recovered to be almost intact (Fig. 4A). TUDCA-treated *Oasis*−/− goblet cells contained abundant PAS-positive mucus in their cytosol. Infiltration of inflammatory cells into the lamina propria was also alleviated in TUDCA-treated *Oasis*−/− mice (Fig. 4A, B). Furthermore, the histological scores of TUDCA-treated *Oasis*−/− mice were significantly decreased compared with those of vehicle-treated mice (Fig. 4C). In addition to the pathological findings, the expression levels of *Bip*, *Chop*, and inflammatory cytokines were significantly reduced in TUDCA-treated *Oasis*−/− mice compared with those in vehicle-treated mice (Fig. 4D–F). The amounts of cleaved caspase-12 and -3, and the number of TUNEL-positive cells in the large intestinal epithelium were notably decreased by administration of TUDCA (Fig. 4G–I). Thus, TUDCA treatment alleviated large intestinal injury in *Oasis*−/− mice exposed to DSS, suggesting that ER stress is the primary cause of DSS-induced colitis in the *Oasis*−/− large intestine.

### Inflammatory responses are induced by ER stress

**Figure 5:**
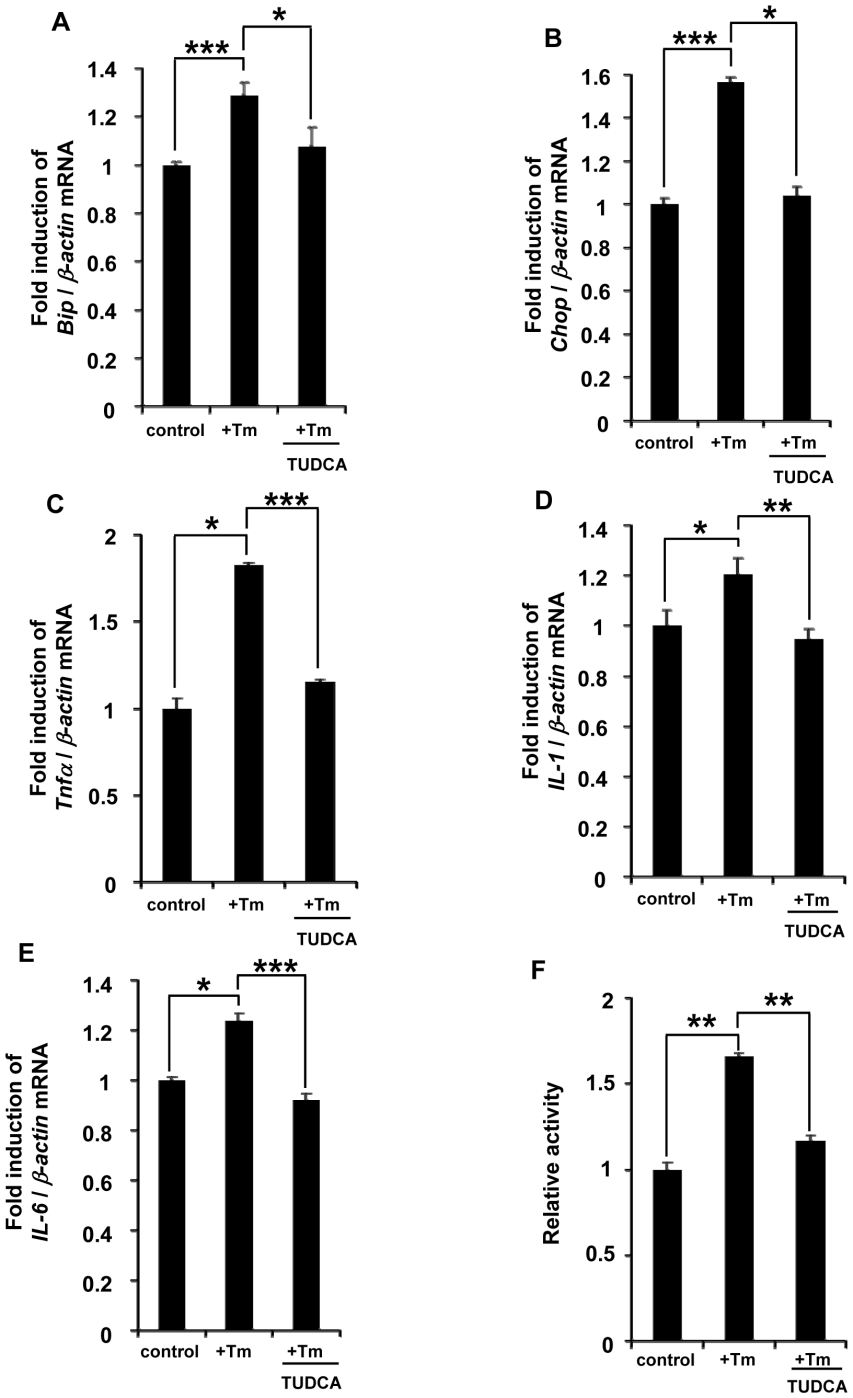
Acceleration of inflammatory responses by ER stress. (A and B) RT-PCR analysis of (A) *Bip* and (B) *Chop* in LS174T human colon carcinoma cells treated with ER stress inducer tunicamycin (Tm) and TUDCA for 24 h (*n* = 4). The expression levels of both *Bip* and *Chop* were downregulated by TUDCA. (C–E) RT-PCR analysis of (C) *Tnfα*, (D) *IL-1*, and (E) *IL-6* in LS174T cells treated with Tm and TUDCA (*n* = 4). Note that the expression levels of these inflammatory cytokines were upregulated in LS174T cells treated with Tm, and downregulated by treatment with TUDCA. (F) Luciferase assay using LS174T cells transfected with the p-Luc reporter plasmid containing the NF-κB binding sequence. Relative activities were increased by treatment of LS174T cells with Tm, and decreased by treatment with TUDCA (*n* = 4). Values represent the means ± s.d. *P<0.05; **P<0.01; ***P<0.001.

ER stress and inflammation were observed in the *Oasis*−/− large intestine exposed to DSS. To analyze the relationship between ER stress and inflammatory responses, we examined the expression of cytokines after ER stress using cell culture models. We used LS174T human colon carcinoma cells in the *in vitro* experiments because it is difficult to culture goblet cells from large intestine of mice and the methods have not been established. LS174T cells were treated with tunicamycin (Tm) to induce ER stress. The expression levels of ER stress markers *Bip* and *Chop*, and inflammatory cytokines *Tnfα*, *IL-1*, and *IL-6* were upregulated in these cells after treatment with Tm for 24 h (Fig. 5A–E). To confirm that the upregulation of cytokines was downstream of the NF-κB pathway, we performed luciferase assay using LS174T cells transfected with a reporter construct including the NF-κB-binding sequence (Fig. 5F). As expected, the reporter activities were significantly elevated in LS174T cells treated with Tm compared with those in untreated cells. TUDCA-treated cells showed a significant reduction in the expression of ER stress markers and inflammatory cytokines, as well as NF-κB activities, suggesting that ER stress promotes inflammation through NF-κB activation.

## Discussion

We found that loss of OASIS function leads to increased susceptibility to DSS-induced colitis based on the following results: 1) *Oasis*−/− mice showed loss of body weight and increased mortality after exposure to DSS; 2) the mucosa of the large intestine in *Oasis*−/− mice showed severe damage involving degeneration and inflammation compared with that in WT mice; 3) ER stress-induced apoptosis was accelerated in the mucosal epithelium of the *Oasis*−/− large intestine. Previously, we reported extreme decrease in the number of mature goblet cells and the amount of mucus in infant *Oasis*−/− mice because of impaired differentiation and maturation of goblet cells [25]. As shown in the present study, we confirmed that the number of mature goblet cells was also decreased in adult *Oasis*−/− mice. It is well known that DSS increases the permeability of the colonic mucosa, followed by induction of mucosal disorders [41]. The mucus abundantly produced by goblet cells composes a mucosal barrier against toxic agents such as DSS. The mucosal epithelial cells of several transgenic mice show reduced production of mucus (*Muc2*−/−, *Winnie*, and *Eeyore* mice) and are susceptible and easily damaged by administration of DSS [27], [30]. Therefore, severe damage of the large intestinal mucosa in *Oasis*−/− mice may be caused by decreased production of mucus in goblet cells that do not mature because of impaired differentiation.

In addition to degeneration and inflammation, ER stress occurred in the mucosa of the large intestine after treatment with DSS. The ER stress in *Oasis*−/− mice was more severe than that in WT mice. Pathological abnormalities in the *Oasis*−/− large intestine were improved by treatment with TUDCA, an agent that alleviates ER stress [37]. This result suggests that ER stress is a crucial factor in exacerbation of tissue damage involving inflammation in the mucosa after administration of DSS. The molecular mechanisms responsible for ER stress induction in the large intestinal mucosa of mice by DSS administration were not elucidated in the present study. It has been reported that inflammatory mediators such as cytokines, chemokines, nitric oxide, and inducible nitric oxide synthase (iNOS) are upregulated in the mucosa of the large intestine with DSS-induced colitis [42]–[44]. In the present study, we showed that some cytokines including *Tnfα* and *IL-1β* were also upregulated in such lesions. TNFα is known to generate reactive oxygen species (ROS) via activation of nicotinamide adenine dinucleotide phosphate (NADPH) oxidase [45], [46]. ROS oxidizes nascent proteins and facilitates accumulation of unfolded proteins in the ER [47]. Furthermore, IL-1β depletes calcium stores in the ER through upregulation of iNOS expression followed by generation of nitric oxide [48]. It is well known that nitric oxide inhibits the function of ER calcium pumps and perturbs Ca^2+^ homeostasis followed by induction of ER stress [49]. Taken together, the ER stress in DSS-induced colitis may be induced by cooperation of these cytokines and inflammatory mediators, and ER stress may be enhanced by the impaired barrier functions of goblet cells in *Oasis*−/− large intestine.

The three major UPR pathways induced by ER stress transducers PERK, IRE1, and ATF6 are known to be involved in inflammatory responses [50]. Translation of IκBα, the major negative regulator for NF-κB, is inhibited by the PERK-eIF2α signaling pathway [51]. Stabilized NF-κB translocates into the nucleus and upregulates the expression of various genes involved in inflammatory responses. The IRE1 signaling pathway directly activates c-Jun N-terminal kinase [52], [53] which is an important mediator for inflammatory signaling and upregulates the expression of inflammatory cytokines. ATF6 activates NF-κB through Akt phosphorylation [54]. In fact, in the *Oasis*−/− large intestinal mucosa, we observed upregulation of inflammatory cytokines, which was probably mediated by these major ER stress transducers. In contrast, inflammatory cytokines also induce ER stress as described above. In this context, ER stress and the induction of inflammatory cytokine expression may be associated with each other to exacerbate inflammation in the *Oasis*−/− large intestine treated with DSS.

In conclusion, differentiation and maturation of intestinal goblet cells are impaired in *Oasis*−/− mice. Severe damage in the *Oasis*−/− large intestine induced by DSS is primarily caused by hypofunction of the mucus barrier. The resultant ER stress and inflammatory responses cooperatively exacerbate the disturbance of the large intestinal mucosa in *Oasis*−/− mice. Thus, OASIS plays crucial roles in protection of the mucosa in the large intestine after injury. Although there is a need for detailed functional analyses of OASIS and its related signaling pathways including the transcriptional targets in patients with IBD, OASIS and its downstream molecules might be novel targets for development of therapeutic strategies against human IBD.

## Supporting Information

Table S1 **The primer sets used for RT-PCR. The following table indicates sets of primers used for RT-PCR.**
(DOC)
