# Supplementary material for: Increased Susceptibility to Dextran Sulfate Sodium-Induced Colitis in the Endoplasmic Reticulum Stress Transducer OASIS Deficient Mice
Source: PLoS One. 2014 Feb 3;9(2):e88048. doi: 10.1371/journal.pone.0088048 (PMC3912207; doi:10.1371/journal.pone.0088048)
Supplement: Table S1 — The primer sets used for RT-PCR. The following table indicates sets of primers used for RT-PCR. (DOC) [file pone.0088048.s001.doc]

**Table S1: Primer sets used for RT-PCR.**

| **Gene name** | **Sense primer (5' - 3')** | **Antisense primer (5' - 3')** |
| --- | --- | --- |
| *OASIS* (mouse) | GAACATGGAGGACTTCTCCAATG | CGGGCTCTGCTCCTGCTTCAC |
| *Tnfα* (mouse) | CATCTTCTCAAAATTCGAGTGACAA | TGGGAGTAGACAAGGTACAACCC |
| *IL-1β* (mouse) | GCAACTGTTCCTGAACTCAACT | GCAACTGTTCCTGAACTCAACT |
| *IL-6* (mouse) | CCAAGAGGTGAGTGCTTCCC | CTGTTGTTCAGACTCTCTCCCT |
| *Tnfα* (human) | CCAGGCAGTCAGATCATCTTC | ATGAGGTACAGGCCCTCTGAT |
| *IL-1β* (human) | CAGATGAAGTGCTCCTTCCAG | TGCACATAAGCCTCGTTATCC |
| *IL-6* (human) | CTGGCAGAAAACAACCTGAAC | CAGGGGTGGTTATTGCATCTA |
| *Bip* (human/mouse) | GTTTGCTGAGGAAGACAAAAAGCTC | CACTTCCATAGAGTTTGCTGATAATTG |
| *Chop*(human/mouse) | GTCCAGCTGGGAGCTGGAAG | CTGACTGGAATCTGGAGAG |
| *β-actin* (human/mouse) | TCCTCCCTGGAGAAGAGCTA | TCCTGCTTGCTGATCCACAT |
